# A case report and literature review of heterotopic mesenteric ossification

**DOI:** 10.1016/j.ijscr.2021.105905

**Published:** 2021-04-27

**Authors:** Raad M.M. Althaqafi, Sara Ahmad Assiri, Rawan Abdulrahman Aloufi, Fawaz Althobaiti, Budur Althobaiti, Mohammad Al Adwani

**Affiliations:** a Department of General Surgery, Al-Hada Armed Forces Hospital, Taif, Saudi Arabia; b College of Medicine, Taif University, Taif, Saudi Arabia

**Keywords:** Case report, Abdominal surgery, Mesenteric ossification

## Abstract

**Introduction and importance:**

Heterotopic mesenteric ossification is a benign bony tissue growth in the mesentery that mostly follows repetitive or severe abdominal injuries leading to reactive bone formation in the mesentery. There are only 73 cases (51 publications) identified in the literature up to the beginning of 2020.

**Case presentation:**

45-year-old Saudi male underwent multiple laparotomies to manage complicated appendicitis which ended with a diverting ileostomy and a colostomy as a mucus fistula. After 9 months, the patient was admitted to the General Surgery department in Al-Hada Armed Forces Hospital for an open ileostomy and colostomy reversal surgery where several irregular bone-like tissues of hard consistency and sharp edges with some spindle-shaped structures resembling needles were found in the mesentery of the small intestine and histopathology revealed of trabecular bone fragments confirming the diagnosis.

**Clinical discussion:**

The majority of cases occur mid to late adulthood with a predilection in the male gender, and usually present with bowel obstruction or an enterocutaneous fistula. Although it has no malignant potential, it may cause severe bowel obstruction that can lead to mortality, it's a rare occurrence and, therefore, is difficult to diagnose among many common abdominal disturbances.

**Conclusion:**

Here we report a rare case of heterotopic mesenteric ossification, which should be considered as one of the delayed complications of abdominal surgery or trauma. The time range of expecting the presentation of heterotopic mesenteric ossification following major abdominal trauma or surgery should be extended and continuously considered during differential diagnosis.

## Highlights

• One of the delayed complications of abdominal surgery or trauma is Heterotopic mesenteric ossification.
• Since 1983 there are only 73 cases of Heterotopic mesenteric ossification published to date.
• Preoperative diagnosis of Heterotopic mesenteric ossification should be based mainly on the characteristic radiographic findings without relying on the levels of calcium or alkaline phosphatase.
• The only way to reach the definitive diagnosis is through excision and histopathological analysis.
• Heterotopic mesenteric ossification has no malignant potential, but it can cause severe bowel obstruction that can lead to mortality in already sick patients.

## Abbreviations

HMO: Heterotopic mesenteric ossification
HO: Heterotopic ossification
CRP: C-reactive protein
ESR: Erythrocyte sedimentation rate
CEA: Carcinoembryonic antigen
CT: Computerized tomography
BMPs: Bone morphogenic proteins

## 1 Introduction

Heterotopic mesenteric ossification (HMO) is a benign bony tissue growth in the mesentery that mostly follows repetitive or severe abdominal injuries leading to reactive bone formation in the mesentery [1]. It is an abdominal catastrophe, and it requires multiple abdominal surgeries to manage. There are only 73 cases (51 publications) identified in the literature up to the beginning of 2020. The pathogenesis of the HMO is currently not well recognized, it is thought to be formed by the stimulation of mesenchymal osteoprogenitor stem cells to differentiate into osteoblasts due to mechanical trauma, ischemia, or intra-abdominal infection [2]. It is also assumed to be caused by implantation of bone periosteum into soft tissue [3].

The majority of cases occur mid to late adulthood with a predilection in the male gender, and usually present with bowel obstruction or an enterocutaneous fistula [4,5]. Although HMO has no malignant potential, it may cause severe bowel obstruction that can lead to mortality in already sick patients [6]. The usual time elapsed from the time of the predisposing trauma to operation ranged from 2 to 4 weeks. However, this might extend to 7 years after the initial insult [1]. Because HMO is a rare occurrence and, therefore, is difficult to diagnose among many common abdominal disturbances, here we present a case of a 45-year-old Saudi male with a typical HMO discovered 9 months after right hemicolectomy in addition to a comprehensive literature review of similar published cases since it was first described in 1983 until 2020.

This work has been reported in line with the SCARE 2020 criteria [7].

## 2 Case presentation

A 45-year-old Saudi male presented to the emergency department of a local hospital in March of 2018 with a typical picture of acute appendicitis; he was admitted for an open appendectomy. Intraoperatively, they discovered a perforated appendix; histopathology revealed a severely inflamed perforated appendix. After 4 days, his first operation was complicated by a feculent discharge from the peritoneal drain due to a complicated cecal fistula with a septic clinical picture. He was admitted for an exploratory laparotomy, and segmental resection of the involved bowel with primary anastomosis was done. Two days after the second operation, he had an anastomotic leak with peritonitis, and he had feculent discharge from the wound site and the peritoneal drain; he was shifted to the operating room for an exploratory laparotomy where a right hemicolectomy was done with primary anastomosis. On the seventh day, and despite the two operative attempts, the patient had intraperitoneal dissemination of fecal material and generalized peritonitis for the third time; he was sent for an exploratory laparotomy where a diverting ileostomy and a colostomy as a mucus fistula was done.

The patient did not have any remarkable family history, he is medically free, not a smoker or alcoholic and doesn't have any significant medical history.

After 9 months, the patient was admitted to the General Surgery department in Al-Hada Armed Forces Hospital for an open ileostomy and colostomy reversal surgery. His abdominal examination revealed a normal soft and lax abdomen with a right ileostomy and left colostomy openings. On admission to Al-Hada Hospital, his white blood cell count was 6.12 × 10^−9^/l, mostly lymphocytes (3.27 × 10^−9^/l). His hemoglobin was 146 g/l, platelet count was 370 × 10^−9^/l. C- reactive protein (CRP) was 1.5 mg/l, erythrocyte sedimentation rate (ESR) was 15 mm/h. Carcinoembryonic antigen (CEA) was 0.9 ng/ml.

White blood cell count normal range is 4 to 11 × 10^−9^/l, lymphocytes normal range is 0.1 to 1.1 × 10^−9^/l. Hemoglobin normal range is 135 to 180 g/l. Platelets normal range is 150 to 400 × 10^−9^/l, C- reactive protein normal range is 0.0 to 5.0 mg/l, erythrocyte sedimentation rate (ESR) normal range is 0.0 to 10.0 mm/h, and Carcinoembryonic antigen (CEA) normal range is 0.0 to 5.0 ng/ml.

**Fig. 1:**
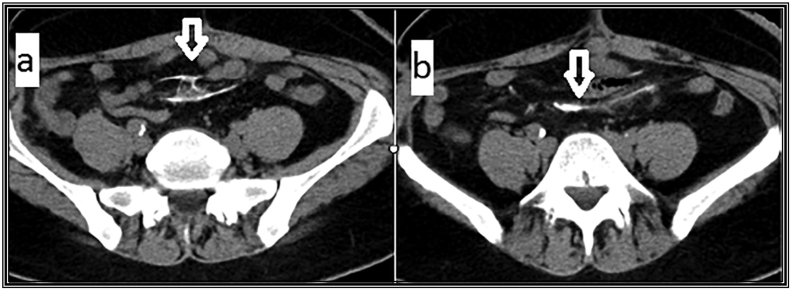
Abdominal computerized tomography (CT) with the contrast. (a) Axial view of the mesentery shows irregular dense calcified shadows (white arrow) not connected to the adjacent bowel surface. (b) Axial view shows ill-defined diffuse fat stranding opacities (white arrow).

**Fig. 2:**
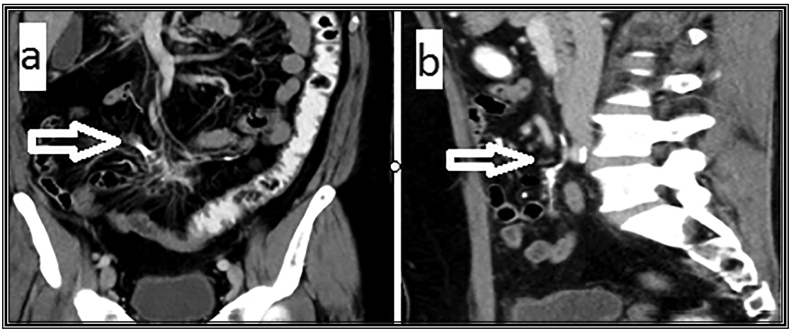
Abdominal computerized tomography (CT) with the contrast. (a) Coronal view of the mesentery shows diffuse focal fat opacification of the mesentery with intervening dense calcified densities (white arrow). (b) Sagittal view shows very thin dense shadows appear longitudinal in position (white arrow) with surrounding mesenteric focal stranding opacity at the site related to previous operations.

Pre-operative abdominal computerized tomography (CT) with the contrast given intravenously, orally, rectally, and through the ileostomy. The axial CT view is shown in (Fig. 1). The coronal and sagittal CT views are shown in (Fig. 2).

**Fig. 3:**
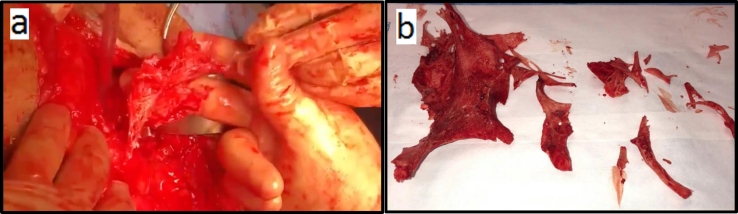
Intra-operative findings (a) A picture of the intraoperative ossified mesentery during the resection. (b) Multiple fragments of hemorrhagic, calcified tissue measuring the largest one (arrow) was 9 × 4.5 × 0.5 cm.

Pre-operative abdominal CT insured a patent passage of the bowel. But the calcified densities and fat stranding opacities were thought to be related to post-operative changes. Intraoperatively, laparotomy under general anesthesia showed adhesions and several irregular bone-like tissues of hard consistency and sharp edges with some spindle-shaped structures resembling needles were found on the mesentery of the small intestine (Fig. 3). All the bone-like tissues were carefully removed.

**Fig. 4:**
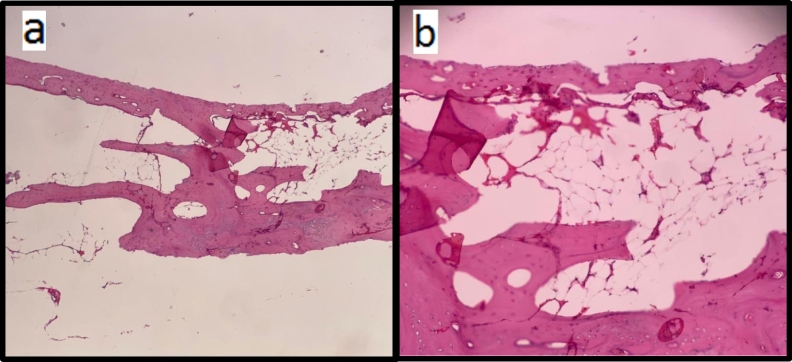
Histopathological analysis of the resected ossified tissue. Histologic appearance of mature heterotopic ossification showing mature trabecular bone fragments with core of fatty marrow cavity. (a) 4× magnification (b) 10× magnification.

The bone-like tissues were examined histologically (Fig. 4). It showed trabecular bone fragments, suggestive of heterotopic ossification. Post-operatively, the patient was advanced slowly to a normal diet, and he improved gradually. The patient's last follow-up was in January 2021; he showed complete recovery with no complications.

## 3 Discussion and conclusion

Heterotopic mesenteric ossification (HMO) was first reported in the literature in 1983, where three patients developed heterotopic mesenteric ossification after abdominal surgery [8,9]. Ectopic calcification is classified histologically into dystrophic calcification (where deposition of calcium happens without osteoblasts) and heterotopic ossification (which differs from dystrophic calcification by the presence of osteoblasts and lamellar bone) [2]. Before 1983, multiple reports of ossification in the abdominal wall due to scars from previous laparotomies were published, and in 1973 a theory was proposed to explain the pathogenesis of abdominal scars heterotopic ossification, which is the differentiation of multipotent embryonic cells [10]. The differentiation of multipotent mesenteric cells as a result of trauma or abdominal surgery can be applied in our case. To date, there is no strong evidence to prove this theory. Another theory was introduced in 1975 in which heterotopic bone formation of laparotomy scars was theorized to result from osteogenic cells deposition from bones adjacent to the scar [11]. Symphysis pubis or xiphoid process irritation during the vertical abdominal incision can lead to periosteal cell implantation, which can be supported by the fact that when horizontal and vertical incisions are made in one patient, the vertical incision is the one that develops calcification [12]. In our case, where the heterotopic ossification developed in the mesentery, this theory can be challenged due to the lack of pre-formed ossified bone around the mesentery.

HMO is extremely difficult to diagnose in patients presenting with abdominal pain and discomfort due to its rare occurrence and very low frequency worldwide. The diagnosis of mesenteric heterotopic ossification can be challenging; abdominal CT scans can help in identifying it preoperatively; however, the differentiation between dystrophic calcification, bone neoplasms, a leakage of contrast, foreign material, or extra-skeletal osteosarcoma from mesenteric heterotopic ossification can be difficult [13]. The only way to reach the definitive diagnosis is through excision and histopathological analysis [14].

We performed an extensive literature search of the Medline and Embase databases for articles published from 1983 up to 2020. No language restrictions were applied, and reference lists of all included studies were manually searched for other potentially eligible studies. We identified only 51 published case reports, including a total of 73 cases. One of whom was an 11-year-old child (Table 1). About (90%) of all the reported cases of mesenteric ossification were males, with a mean age of 48.38 ± 18.27; the most common presenting symptom was bowel obstruction (41%). About (16.4%) of the cases were discovered incidentally by imaging, while (13.7%) of the cases were discovered during surgery. Most (80%) of the reported cases had a surgical history of laparotomy, and (71.2%) of the ossification developed in the mesentery. Detailed statistical analysis of all reported cases is shown in (Table 2). The current case is in line with the majority of HMO cases, with a history of abdominal surgery that has preceded the formation of HMO.

**Table 1:** Literature review summary.

Year	Authors	Age	Gender	Surgical History	Presenting symptoms	Site
1983	Hanesn et al. [8]	55	Male	Coloproctectomy for severe ulcerative colitis	Bowel obstruction	Mesentery
	Lemeshev et al. [9]	44	Male	Laparotomy for small bowel obstruction	Bowel obstruction	Mesentery
1989	Myers et al. [2]	57	Male	resection of the right colon followed by exploratory laparotomy and removal of tumor from the celiac plexus.	Bowel obstruction	Mesentery
1992	Yannopoulos et al. [24]	63	Male	Aortic bifemoral bypass & and two laparotomies	Bowel obstruction	Mesentery & omentum
1999	Wilson et al. [25]	75	Male	Repair of an abdominal aortic aneurysm	Bowel obstruction	Mesentery
		76	Male	Left hemicolectomy for adenocarcinoma and repair of an abdominal aortic aneurysm	Bowel obstruction	Mesentery
		43	Male	No Surgical History	Bowel obstruction	Mesentery
		80	Male	No Surgical History	Bowel obstruction	Mesentery
		43	Male	Laparotomy for incarcerated umbilical hernia	Bowel obstruction	Mesentery
2000	Marucci et al. [26]	25	Male	Laparotomy	Bowel obstruction	Mesentery
2001	Hakim et al. [27]	50	Male	Nephrectomy and left colon resection with a colostomy	Enterocutaneous fistulas	Mesentery
2003	Lai et al. [28]	60	Male	Emergent laparotomy with total colectomy and end ileostomy	Mass & discomfort in the peri-ileostomy region	Mesentery
2004	Bovo et al. [29]	76	Male	No Surgical History	Bowel obstruction	Mesentery
	Compérat et al. [30]	64	Male	3 Laparotomies for intestinal obstruction and adhesiolysis	Bowel obstruction	Mesentery
		76	Female	Right hemicolectomy	Bowel obstruction	Mesentery & omentum
2005	Tonino et al. [13]	39	Male	Abdominal gunshot injury managed Laparotomy with partial resection of small bowel and colon, and construction of a temporary ileostomy followed by Laparotomy for enterocutaneous fistulae	No symptoms discovered incidentally during surgery to reverse the ileostomy	Mesentery
	Androulaki et al. [31]	74	Male	Reconstruction of an umbilical hernia and cholecystectomy and Prostatectomy	Bowel obstruction & and mild renal failure	Mesentery
	Kao et al. [32]	60	Male	Hartmann procedure with ileostomy for treatment of diverticulitis	No symptoms discovered incidentally by imaging	Mesentery
2006	Gouëllo et al. [33]	25	Male	Blunt abdominal Trauma followed by 50-cm distal ileum resections and a temporary ileostomy	Bowel obstruction	Mesentery
	Patel et al. [6]	51	Male	4 of them had significant abdominal surgery 1 past trauma 1 with no Surgical History	Bowel obstruction + peritonitis	Mesentery & omentum
		21	Male		Bowel obstruction	Omentum
		65	Male		Bowel obstruction	Mesentery
		62	Male		Bowel obstruction	Mesentery
		22	Male		Bowel obstruction	Mesentery
		72	Male		Bowel obstruction	Mesentery
	Zamolyi et al. [34]	43	Male	Almost all had previous abdominal surgery	N/A	Omentum
		32	Female		N/A	Omentum
		37	Male		N/A	Mesentery
		24	Male		N/A	Mesentery
		68	Male		N/A	Mesoappendix
		47	Male		N/A	Colon
2007	Ibáñez Alonso et al. [35]	64	Male	Multiple laparotomies to manage hemorrhagic colitis.	Abdominal pain and stiffness	Mesentery
2008	Como et al. [36]	51	Male	Abdominal gunshot injury managed Laparotomy and transverse colon resection with end colostomy, then re-explored again due to extensive necrosis then developed and abdominal fistula	Bowel obstruction	Mesentery
	Jacob et al. [37]	26	Male	Post Blunt abdominal trauma and Laparotomy for abdominal compartment syndrome, distal ileum and ascending colon were resected due to intestinal ischemia.	No symptoms discovered incidentally by imaging	Mesentery
2009	Vlachos et al. [38]	42	Male	Two Laparotomies due to massive hematemesis with total gastrectomy with a Roux-en-Y oesophagojejunal anastomosis	Uncontrollable septic fever,	Omentum
	Abensur et al. [39]	67	Female	Uterine leiomyoma removal Urolithiasis and two cesarean sections	Dysuria, urinary incontinence and nocturia	Mesentery involving pelvis
	Hayashi et al. [40]	40	Male	Exploratory laparotomy twice for suspect intraperitoneal hemorrhage and small bowel resection	Bowel obstruction	Mesentery
2010	Yushuva et al. [41]	2 cases	–	Gastric bypass with Roux-en-Y reconstruction procedure for morbid obesity and subsequently presented with gastrointestinal fistulae	–	Mesentery
2011	Shi et al. [42]	39	Male	Left hemicolectomy was performed for the treatment of descending colon adenocarcinoma	Bowel obstruction	Omentum
	Reynoso et al. [43]	59	Female	Complicated gynecologic laparoscopic oophorectomy, abdominal sepsis, multiple small-bowel resections, and skin grafting for an open abdomen	Persistent enterocutaneous fistula	Mesentery
2012	Baker et al. [44]	29	Female	Abdominal gunshot wound managed by right hemicolectomy, right nephrectomy, Whipple procedure with pancreatic and duodenal resection, repair of inferior vena cava, and provisional ostomy in the midline abdominal wound	No symptoms discovered incidentally by imaging	Mesentery & omentum
		37	Male	Abdominal gunshot wound managed by damage control laparotomy and bowel resection for liver and spleen lacerations, multiple enterotomies, and mesenteric injury	No symptoms discovered incidentally by imaging	Mesentery & Omentum
		19	Male	Abdominal gunshot wound with small bowel injury managed by damage-control laparotomy and bowel resection	No symptoms discovered incidentally by imaging	Mesentery & Omentum
		62	Male	Bladder cancer managed by cystoprostatectomy and lymph node dissection with ileal conduit; this was complicated by anastomotic breakdown and small bowel ischemia requiring additional laparotomies	No symptoms discovered incidentally by imaging	Mesentery & Omentum
		31	Male	Splenic, diaphragmatic, colonic, and small bowel injuries sustained in a motor vehicle collision, requiring partial colectomy	No symptoms discovered incidentally by imaging	Mesentery & Omentum
	Ioannidis et al. [45]	25	Male	Splenectomy and open cholecystostomy	Esophagotracheal fistula	Peritoneum
2013	Jhanwar et al. [46]	11	Male	No Surgical History	Bowel obstruction	Mesentery
	Torgersen et al. [14]	58	Male	Intestinal resection due to perforated diverticulitis, then the patient developed an enterocutaneous fistula	Enterocutaneous fistula	Mesentery
	Ma et al. [12]	53	Male	Emergency temporary ileostomy for the hemorrhagic Meckel's diverticula with anastomotic fistula following right hemicolectomy	Discovered incidentally in the OR	Mesentery
	Nabulyato et al. [47]	48	Male	Emergency cecostomy and loop ileostomy procedures for peritonitis secondary to “spontaneous” sigmoid colon perforation	Discovered incidentally in the OR	Mesentery
2014	Honjo et al. [48]	88	Male	Abdominal aortic repair, followed by a second operation for an ileus tube insertion into the jejunum	Bowel obstruction	Mesentery
	Caitlin et al. [49]	32	Male	Stab wound to the abdomen requiring exploratory laparotomy with small bowel resection	Intermittent abdominal pain	Mesentery
	Obeid et al. [17]	36	Male	Bullet injury to the abdomen and multiple subsequent laparotomies, complicated by a complex abdominal wall hernia with enterocutaneous fistulae	Vague abdominal discomfort and foul-smelling discharge from abdominal wall defect	Mesentery
	Nerup et al. [50]	64	Male	Blunt abdominal trauma, colectomy with primary anastomosis	Discovered incidentally in the OR during stoma reversal	Mesentery
2015	Bakoš et al. [51]	30	Male	Four Abdominal Surgeries	Discovered incidentally in the OR	Mesentery
	Schiergens et al. [52]	34	Male	Colonic perforation with severe fecal peritonitis followed by a Hartmann procedure	No symptoms discovered incidentally by imaging	Facia and mesentery
	Christopher Vytlacil et al. [53]	58	Male	Sigmoid colectomy for stage 2 colon adenocarcinoma	Bowel obstruction	Mesentery
	Nashed et al. [1]	24	Male	Sigmoid resection followed by another surgery of transverse colostomy	Enterocutaneous fistulas	Mesentery
	Penev et al. [54]	49	Male	Numeral exploratory laparotomies performed after a blunt abdominal trauma	No symptoms discovered incidentally by imaging	Mesentery
2016	Herrera-Toro et al. [55]	14	Male	Neonatal colostomy and then posterior sagittal anorectoplasty. In addition to a Surgical decompression of tethered spinal cord syndrome.	Bowel obstruction	Mesentery
	Mussato et al. [56]	55	Male	Sigmoid colon resection and washout due to perforated sigmoid diverticulitis and fecal peritonitis	Discovered incidentally in the OR	Mesentery
		60	Male	Exploratory laparoscopy for intestinal obstruction,	Bowel obstruction	Mesentery
	Sapalidis et al. [57]	81	Male	Sigmoidectomy	Discovered incidentally in the OR	Mesentery
	Georgios Sahsamanis et al. [58]	55	Male	Hartmann's colostomy	Discovered incidentally in the OR	Mesentery & pertoniem
	Sun et al. [59]	35	Male	Hartmann's procedure followed by delayed abdominal closure	Palpable, and nonpainful abdominal masses.	Mesentery
2017	Ferreira et al. [5]	45	Male	Segmental enterectomy and temporary ileostomy and subsequent multiple surgeries with small bowel resection	No symptoms discovered incidentally by imaging	Mesentery and abdominal wall
		60	Male	Radical right colectomy for a malignant condition, complicated with anastomotic dehiscence and septic shock, which led to anastomosis take down and vacuum pack laparostomy	Discovered incidentally in the OR	Mesentery and abdominal wall
2018	Matthew Amalfitano et al. [60]	70	Male	Hemicolectomy for adenocarcinoma	Post-mortem examination	Mesentery
	Michael et al. [61]	34	Male	Laparotomy for Grade III liver injury and pancrea9c tail lacera9on complicated with transverse colon perfora9on and duodenal stump leak	No symptoms discovered incidentally by imaging	Mesentery
2019	Bosaily et al. [62]	52	Male	Ileostomy	Discovered incidentally in the OR	Stomal site
	Çelik et al. [63]	41	Male	Emergency right nephrectomy, right hemicolectomy with end ileostomy, and applications of intraabdominal vacuum-assisted closure therapy 15 months prior to admission.	No symptoms discovered incidentally by imaging	Mesenteric, omental, and peritoneal
2020	Andrea Aurelio et al. [64]	28	Male	No surgical history, patient had a history of blunt thoracic and abdominal trauma	Bowel obstruction	Mesentery

**Table 2:** Statistical analysis of all reported cases in the literature.

Parameters	Total reported cases (73)
Age mean (SD)	48.38 ± 18.27

Gender	
Male n (%)	66 (90.4)
Female n (%)	5(6.8)
Not mentioned n (%)	2(2.7)

Surgical\trauma history	
Laparotomy n (%)	59(80.8)
Laparotomy due to a gunshot wound n (%)	4(5.5)
Laparotomy due to trauma n (%)	3(4.1)
Trauma n (%)	2(2.7)
No surgical history n (%)	5(6.8)
Not mentioned n (%)	

Clinical presentation	
Bowel obstruction n (%)	30(41.1)
Mass n (%)	2(2.7)
Peritonitis n (%)	1(1.4)
Enterocutaneous fistula n (%)	5(6.8)
UTI symptoms n (%)	1(1.4)
Fever n (%)	1(1.4)
Esophagotracheal fistula n (%)	1(1.4)
Abdominal pain n (%)	1(1.4)
Incidental in the OR n (%)	10(13.7)
Incidental in the imaging n (%)	12(16.4)
Incidental in the postmortem autopsy n (%)	1(1.4)

Ossification site	
Mesentery n (%)	52(71.2)
Omentum n (%)	5(6.8)
Mesentery and omentum n (%)	8(11)
Mesoappendix n (%)	1(1.4)
Colon n (%)	1(1.4)
Mesentery and peritoneum n (%)	1(1.4)
Mesentery and abdominal bowel n(%)	3(4.1)
Mesentery and peritoneum and omentum n (%)	1(1.4)
Stomal site n (%)	1(1.4)

The time that passed from the last surgical operation to the intraoperative discovery of HMO in the current case was 9 months. The time required for the formation and appearance of HMO clinical symptoms is not exactly known but ranged from 2 weeks to 2 years [15]. Although HMO is rarely encountered, due to the increased cases reported in the last decade, it should be considered in the differential diagnosis in patients presenting with intestinal obstruction or if dense calcified shadows were observed on abdominal CT in patients who underwent previous abdominal trauma or surgeries.

Bone morphogenic proteins (BMPs) are multifunctional cytokines that are a part of the transforming growth factor-β family released from inflammatory cells at the site of inflammation, injury, wounds, or sepsis, and have been reported to stimulate the formation of abnormal cartilage and bone tissues [16,17]. BMP and its signalling were observed to be increased in experimental models of trauma-induced heterotopic ossification (HO); meanwhile, BMP antagonism has been shown to decrease HO expansion. Anticipated HO formation after abdominal surgical operations was prevented by the use of anti-inflammatory [18]. Interestingly, rapamycin, which decreases inflammatory signalling through inhibition of the mTOR mechanism of activation, was reported to alleviate HO formation [19]. Moreover, the levels of both local and systemic inflammatory markers were suggested to be increased in traumatic HO as there is a positive correlation between inflammatory cytokines levels and the likelihood of HO formation [20].

In our case, the patient was admitted with severe abdominal pain that reoccurred with each complication and necessitated multiple surgeries. This pain is sensed by substance P, a member of the tachykinin peptide family, that was demonstrated to transmit nociceptive sensation *via* primary sensory fibres to the spine and brainstem [21]. This substance P was demonstrated to increase and mediate BMP-dependent HO formation [22]. The serum level of substance P is elevated in HO patients, and serum from neurogenic HO mice was demonstrated to induce osteogenic transformation of mesenchymal progenitor cells *in vitro* [23].

**Fig. 5:**
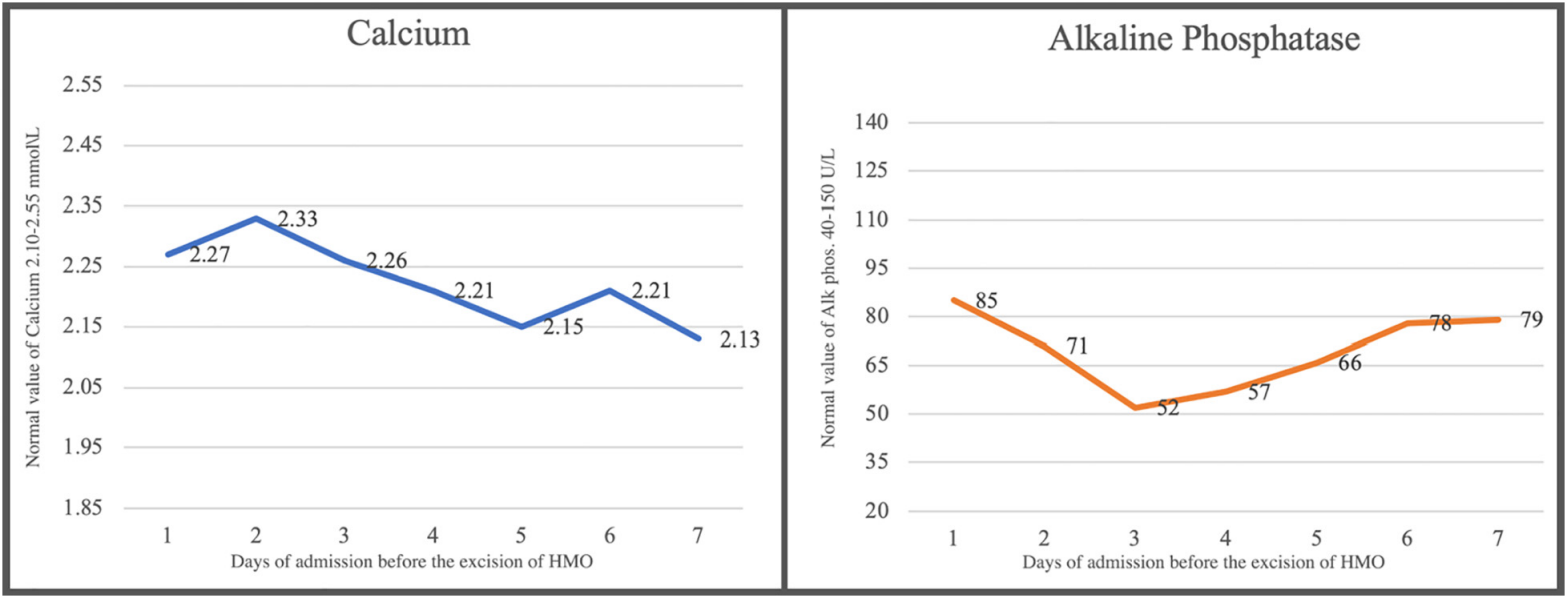
Calcium and alkaline phosphatase progression. The progression of calcium and alkaline phosphatase levels during our case's admission and before discovering the ossified mesenteric bones intraoperatively. All readings were within normal suggesting that a mature ossified bone fragments are already formed.

Mesenteric ossification can recure after the removal of the mesenteric bony fragments surgically; calcium and alkaline phosphatase levels can predict the recurrence. If the patient had a low calcium level and a high alkaline phosphatase level, which might indicate an ongoing process of osteogenesis and an active osteoblast [2]. Our patient had normal calcium and alkaline phosphatase levels preoperatively (Fig. 5), suggesting that mature ossified bones has already been formed, which is confirmed by histopathology.

Among the 52 HMO cases presented in the literature, only five cases showed elevated levels of alkaline phosphatase, of which four cases presented 3 weeks after the predisposing trauma or surgery while the patient in the current case was admitted 9 months after the inciting operation. This might indicate the vast variation in the speed of the HMO pathogenesis from case to case, which might be attributed to the levels of inflammation during and after the surgeries, amount of released cytokine, and the ability of the body to control and adjust the orchestra of inflammation. Moreover, the pathogenesis of the HMO might be accelerated or delayed depending on the post-operative management of the case, as precise management through proper anti-inflammatory drugs might prevent or delay the pathogenesis course of the HMO. Additionally, the delayed formation of the HMO, as we encountered in the current case, might indicate the need for long-time management with continuous monitoring of the serum inflammatory cytokines even after the subside of the pain associated with the surgical operation as to continue controlling the inflammatory milieu to avoid delayed HMO formation.

## 4 Conclusion

In summary, here we report a rare case of HMO, which should be considered as one of the delayed complications of abdominal surgery or trauma. The time range of expecting the presentation of HMO following major abdominal trauma or surgery should be extended and continuously considered during differential diagnosis, especially when there is a history of previous surgery or trauma. Diagnosis of HMO should be based mainly on the characteristic radiographic findings without relying on the level of alkaline phosphatase, which is elevated only in the period of active osteogenic stag. Continuous monitoring and controlling of the inflammatory cytokines not only for a short time post-operatively but for an extended period may prevent or delay the HMO formation.

## Sources of funding

No funding was received.

## Ethical approval

The study was approved by the Research Ethics Committee at Al-Hada Armed Forces Hospital and is available upon request from the corresponding author. (reference number, 19200).

## Consent

Written informed consent was obtained from the patient for publication of this case report and accompanying images. A copy of the written consent is available for review by the Editor-in-Chief of this journal on request.

## Research registration (for case reports detailing a new surgical technique or new equipment/technology)

Not applicable.

## Guarantor

Sara Ahmad Assiri

Taif University School of Medicine Taif, Saudi Arabia

saraassiriiii@gmail.com

Al Qutbiyyah AT TAIF Kingdom of Saudi Arabia

## Provenance and peer review

Not commissioned, externally peer-reviewed.

## CRediT authorship contribution statement

Sara Assiri and Raad Althaqafi led the writing of the case report and literature review, Rawan Aloufi, Fawaz Althobaiti, Budur Althobaiti, and Mohammad Al Adwani assisted with writing and revision of the manuscript All authors read and approved the final manuscript.

## Declaration of competing interest

No conflict of interest.
